# Characterization of an Aminopeptidase A from *Tetragenococcus halophilus* CY54 Isolated from Myeolchi-Jeotgal

**DOI:** 10.4014/jmb.2210.10003

**Published:** 2023-01-04

**Authors:** Tae Jin Kim, Min Jae Kim, Yun Ji Kang, Ji Yeon Yoo, Jeong Hwan Kim

**Affiliations:** 1 Division of Applied Life Science (BK21 Four), Graduate School, Gyeongsang National University, Jinju 52828, Republic of Korea; 2 Institute of Agriculture and Life Science, Gyeongsang National University, Jinju 52828, Republic of Korea

**Keywords:** *Tetragenococcus halophilus*, aminopeptidase A, *pepA* gene, proteolytic system

## Abstract

In this study, a *pepA* gene encoding glutamyl (aspartyl)-specific aminopeptidase (PepA; E.C. 3.4.11.7) was cloned from *Tetragenococcus halophilus* CY54. The translated PepA from *T. halophilus* CY54 showed very low similarities with PepAs from *Lactobacillus* and *Lactococcus* genera. The *pepA* from *T. halophilus* CY54 was overexpressed in *E. coli* BL21(DE3) using pET26b(+). The recombinant PepA was purified by using an Ni– NTA column. The size of the recombinant PepA was 39.13 kDa as determined by SDS-PAGE, while its optimum pH and temperature were pH 5.0 and 60°C, respectively. In addition, the PepA was completely inactivated by 1 mM EDTA, indicating its metallopeptidase nature. The *K*m and *V*max of the PepA were 0.98 ± 0.006 mM and 0.1 ± 0.002 mM/min, respectively, when Glu-*p*NA was used as the substrate. This is the first report on PepA from *Tetragenococcus* species.

## Introduction

Microorganisms belonging to the *Tetragenococcus* genus are halophilic lactic acid bacteria (LAB) that are often isolated from fermented foods with high salinities, such as jeotgal [1], fish sauce [2], soy sauce [3] and doenjang [4]. *Tetragenococcus* spp. can grow in the presence of 20% (w/w) NaCl and grow rapidly at 20-30°C. Due to their high salt tolerance and status as members of LAB, *T. halophilus* strains have been tested as a starter for fish sauce and soy sauce fermentations as they can accelerate fermentation and add flavor to products [5, 6]. Strong proteolytic activity is an essential requirement for a strain if it is to be used as a starter for fermented foods. Such strains can grow rapidly in foods rich in proteins and generate sufficient amounts of peptides and amino acids over time, which contributes to the development of flavor and functionalities of the products [7, 8]. However, the biochemical and genetic underpinnings of the proteolytic systems of *T. halophilus* have not been studied. For the utilization of exogenous proteins as nitrogen sources, bacteria including LAB produce several different types of proteases and peptidases, and some of them are secreted into culture medium. Proteases and peptidases are classified into two groups, endo- and exo-type enzymes. Both types of enzymes are required for the efficient hydrolysis of proteins, and the combined actions of both types of enzymes enable quick conversion of large-sized proteins into peptides and amino acids [7]. In this respect, understanding the proteolytic systems of *T. halophilus* is desirable in evaluating *T. halophilus* strains for industrial applications, such as their potential as starters for fermented foods including jeotgal, fish sauce, and soy sauce.

PepA (aminopeptidase A) is a metal-dependent exopeptidase specific for peptides with glutamic acid, aspartic acid or serine at the N-terminus [9]. PepA has potential applications in the hydrolysis of food proteins such as gluten and casein, which are rich in glutamate and aspartate [10, 11]. PepA hydrolysis of glutamate-rich proteins is desirable because the released glutamate confers umami taste [12]. In this work, we report for the first time the purification and characterization of PepA from *T. halophilus* CY54, an isolate from a jeotgal product. We compared the PepA from *T. halophilus* CY54 with other LAB PepAs previously reported. Our results should help to better understand and evaluate the proteolytic capacity of *T. halophilus* strains while also underscoring the importance of these strains in industrial applications.

## Materials and Methods

### Cloning of *pepA* from *T. halophilus* CY54

pepA was amplified by PCR from the genome of *T. halophilus* CY54 [13]. The primer pair used was: *pepA*F (5'-ACTGGATCCTACGATCAAACTTGC-3', BamHI site underlined) and *pepA*R (5'-TTACTCGAGTTTTCC CGCAATAATCG-3', XhoI site underlined). Amplification conditions were as follows: 94°C for 1 min; 30 cycles of 94°C for 30 s, 59°C for 30 s, 72°C for 1 min; and final extension at 72°C for 4 min. The amplified product was ligated with pGEM T-Easy vector (Promega, USA). *E. coli* DH5α cells were transformed with the ligation mixture by electroporation. Plasmid DNA was prepared from an *E. coli* transformant (TF) and the insert was sequenced at Cosmogenetech (Korea).

### Overexpression of *pepA* in *E. coli* and Purification of Recombinant PepA

pepA was amplified as described above and ligated with pET26b(+) (Novagen, USA) (5.36 kb, Kan R). The ligation mixture was used to transform *E. coli* BL21(DE3) cells by electroporation, and a TF harboring pETA54 (pET26b(+) with *pepA*) was obtained. LB broth (100 ml) containing kanamycin (60 μg/ml) was inoculated with the TF and the culture was grown at 37°C until the OD600 reached 0.6. Then, isopropyl β-D-1-thiogalactopyranoside (IPTG) was added to 0.5 mM concentration. After 12 h of growth at 20°C, cells were harvested by centrifugation at 12,000 ×*g* for 15 min at 4°C, washed three times with phosphate-buffered saline (PBS, pH 7.4), and resuspended in lysis buffer (50 mM NaH_2_PO_4_, 300 mM NaCl, and 10 mM imidazole, pH 7.0). Cells were then disrupted by sonication (Ultrasonicator, Bandelin Electronic, Germany) and centrifuged at 12,000 ×*g* for 15 min. The resulting pellet (insoluble fraction) and supernatant (soluble fraction) were examined for PepA by SDS-PAGE. The soluble fraction was loaded onto a Ni-NTA column (GE Healthcare, Sweden). Bound PepA was eluted from the column by stepwise increase in the imidazole concentration (40−500 mM) of the elution buffer. Protein concentration was determined by Bradford method using a BioRad protein assay kit [14]. SDS-PAGE was done using a 10% (w/v) acrylamide gel for separation and a 5% (w/v) gel for stacking.

### Enzyme Assay and Properties of Recombinant PepA

The activity of the recombinant PepA was measured by a method previously reported [15], and glu-*p*NA (Sigma-Aldrich, USA) was used as the substrate. The reaction mixture (500 μl) consisted of 50 μl enzyme solution (10 μg PepA in 50 μl PBS), 400 μl 100 mM potassium phosphate buffer (pH 7.0), and 50 μl 10 mM substrate. The mixture was incubated at 40°C for 30 min, and then 1 ml 30% acetic acid was added to stop the reaction. The absorbance at 410 nm was measured using a spectrophotometer (UV-1601, Shimadzu, Japan). A standard curve was prepared using various concentrations of *para*-nitroaniline. One unit of aminopeptidase activity (U) was defined as the amount of enzyme that released 1 nmol of *p*-nitroaniline in 1 min under the assay conditions.

The effect of pH on the PepA activity was examined. First, purified PepA (10 μg) was incubated for 1 h at 40°C at pH 3-9 and the remaining activities were measured as described above. Buffers of 100 mM concentration were used: citrate-NaOH (pH 3-6), potassium phosphate (pH 6-8), and borate-NaOH (pH 8-9). The effect of temperature on the PepA activity was then determined after 1 h incubation at 20-90°C (pH 5.0). The effects of NaCl concentration (0-20%), metal ions (2 mM) and protease inhibitors were also determined after 1 h incubation at 60°C and pH 5.0 (100 mM citrate-NaOH buffer). The substrate specificity of PepA was determined with different chromogenic substrates including Glu-*p*NA, Met-*p*NA, Ala-*p*NA, Arg-*p*NA, Lys-*p*NA, and Pro-*p*NA at 60°C and pH 5.0 (100 mM citrate-NaOH buffer). The kinetic parameters of the recombinant PepA were determined at 60°C and pH 5.0 (100mM citrate-NaOH buffer) with Glu-*p*NA (0.1-2 mM) as the substrate.

### Heterologous Expression of *pepA* in *Weissella confusa*

pepA was amplified by using the primer pair: F2 (5`-CCCGGATCCTGCTGCTTCATAATG-3`, BamHI site underlined) and R2 (5`-TTTCTGCAGCTGGCAACCTCAATC-3`, PstI site underlined). Amplified *pepA* was ligated with pHG240E (5.9 kb, EmR) after the BamHI and PstI digestions. pHG240E is a *Lactobacillus*-*E. coli* shuttle vector based on a 1.8 kb cryptic plasmid, pHG1, from *Levilactobacillus zymae* GU240 [16], and an *E. coli* vector, pBluescriptII KS(+) (2.9 kb, AmpR). The construction of pHG240E will be described elsewhere (manuscript in preparation). The ligation mixture was used to transform *E. coli* DH5α competent cells. pHG240EpepA (pHG240E with *pepA*) was prepared from an *E. coli* transformant and used to transform *Weissella confusa* CB1 by electroporation. The PepA activities of the TFs were measured as described above.

## Results and Discussion

### Cloning of *pepA* from *T. halophilus* CY54

*T. halophilus* CY54 was isolated from myeolchi-jeotgal in 2020 [13]. Among more than 1,000 LAB isolates, *T. halophilus* CY54 was selected because of its high proteolytic activities on MRS agar plates with skim milk (2%, w/v). *T. halophilus* CY54 showed desirable properties as a starter for fermented foods with high salinities, which included high proteolytic activity, high salt tolerance, no production of biogenic amines, and sensitivity to medically important antibiotics. The whole genome sequence of *T. halophilus* CY54 was determined and sequence analyses showed that *T. halophilus* CY54 possessed 21 putative protease genes and 14 aminopeptidase genes [13]. Among them, a *pepA* gene encoding glutamyl (aspartyl)-specific aminopeptidase (PepA; E.C. 3.4.11.7) was chosen for further studies. PepA is an exopeptidase which specifically cuts peptides with the N-terminal amino acids aspartic acid and glutamic acid [9]. Peptidases such as PepA contribute to the overall proteolytic capacity of LAB strains and help host cells grow in environments containing proteins such as casein and wheat gluten, both of which are rich in glutamyl/aspartyl [17].

**Fig. 1:**
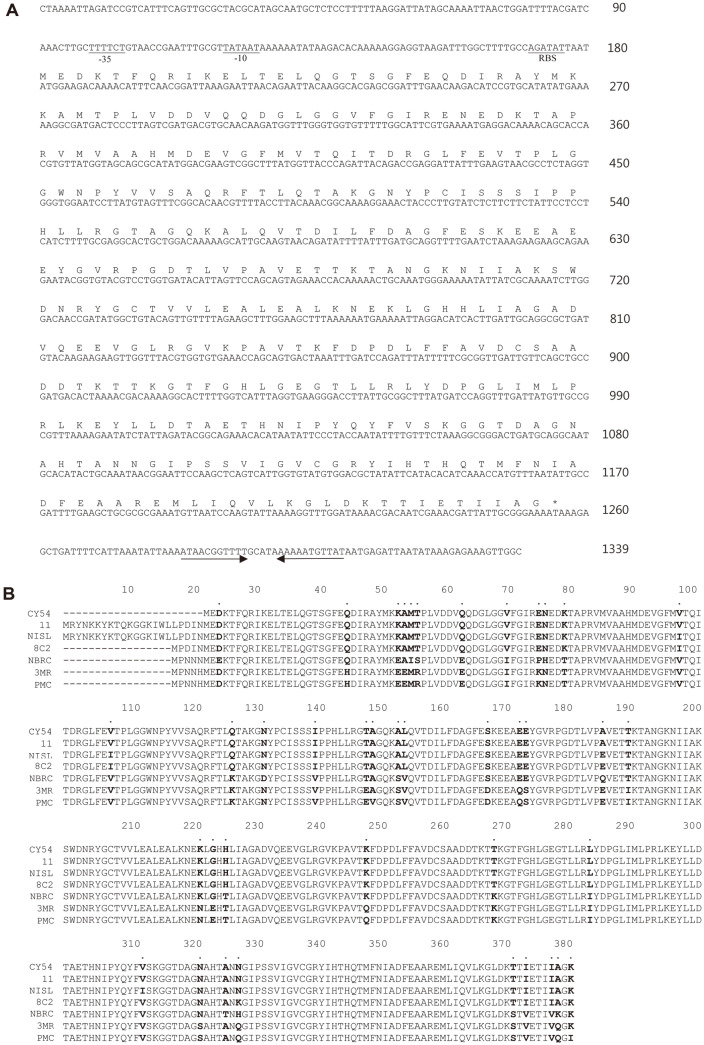
Nucleotide and translated amino acid sequences of *pepA* from *T. halophilus* CY54. (**A**) Nucleotide and translated amino acid sequences. Putative -10 and -35 promoter sequences, and RBS are underlined. The two horizontal arrows indicated a putative transcription terminator. (**B**) Alignment of amino acid sequence of CY54 PepA with other PepAs. 11, *T. halophilus* 11; NISL, *T. halophilus* NISL 7116; 8C2, *T. halophilus* 8C2; NBRC, *T. koreensis* NBRC 106072; 3MR, *T. muriaticus* 3MR10-3; PMC, *T. muriaticus* PMC-11-5. Amino acids showing difference are marked as bold and dotted at the top.

A 1.1 kb *pepA* gene was amplified, and the nucleotide sequence was determined. An ORF of 1,077 nucleotides that could encode a protein of 358 amino acids was located and had a calculated size of 39.13 kDa and an isoelectric point (pI) of 5.28 (Fig. 1A). When the translated amino acid sequence of the PepA from *T. halophilus* CY54 (CY54 PepA) was aligned with the reported PepAs, CY54 PepA showed the highest similarity to PepA from *T. halophilus* 11 (GBD61355) (100%) [18], followed by *T. halophilus* NISL 7116 (GBD65982) (98.8%) [19], *T. halophilus* 8C2 (GEQ38298) (98.8%) [20], *T. koreensis* NBRC 106072 (GEN92074) (93.3%) [21], *T. muriaticus* 3MR10-3 (91.6%) [22] and *T. muriaticus* PMC-11-5 (KFN93504) (91.32%) [22] (Fig. 1B). These PepAs from *Tetragenococcus* strains have not been characterized yet and their presence was expected based on annotation of the genome sequences. CY54 PepA showed very low similarities to those from *Lactobacillus delbrueckii* subsp. *lactis* DSM 20072 (13.4%) [17] and *Lactococcus lactis* subsp. *lactis* MG1363 (21.7%) [23], which have been characterized and studied extensively in the past. The results clearly show that CY54 PepA, and most likely other PepAs from *Tetragenococcus*, are significantly different from those from other LAB genera. The GenBank accession number for CY54 PepA is QXN87940.

### Overexpression of *pepA* in *E. coli* BL21(DE3) and Purification of Recombinant PepA

**Fig. 2:**
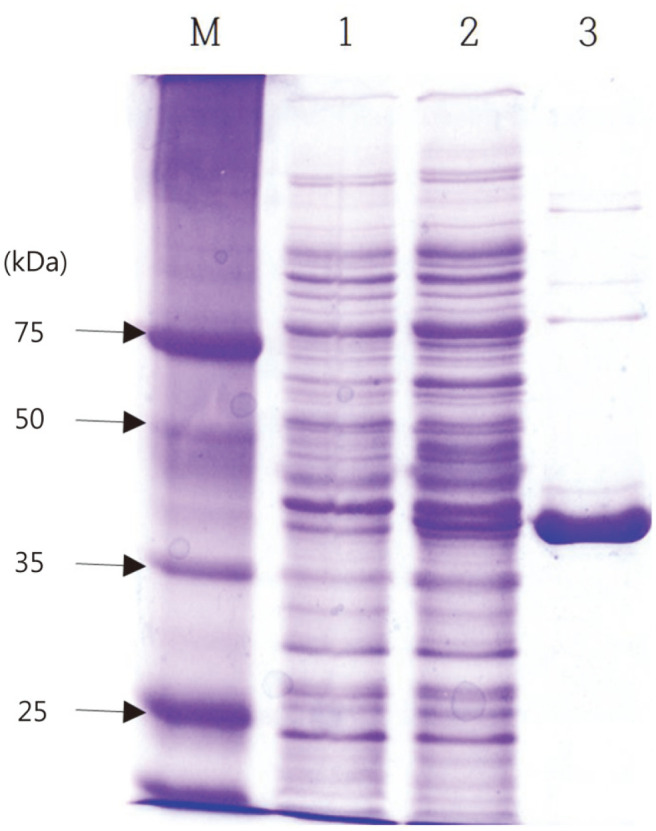
SDS-PAGE of recombinant PepA. M, size marker (PagePuler unstained broad range protein ladder, Thermo Fisher scientific, Waltham, MA, USA); 1, soluble fraction from *E. coli* BL21 (DE3) [pET26(b)+]; 2, soluble fraction from *E. coli* BL21 (DE3) [pETA54]; 3, PepA eluted from a Hi-Trap affinity column at 100 mM imidazole concentration.

The *pepA* gene from *T. halophilus* CY54 was overexpressed in *E. coli* BL21(DE3) and recombinant PepA was produced in IPTG-induced cells (Fig. 2, lane 2). A control, *E. coli* BL21(DE3) harboring intact pET26b(+), did not produce PepA (Fig. 2. lane 1). PepA was observed from both soluble and insoluble fractions of cell extract (results not shown). The soluble fraction was used to purify the recombinant PepA since active enzyme was likely present in the soluble fraction rather than in the insoluble fraction. The soluble fraction was loaded onto a Ni-NTA column, and bound PepA was eluted at 100 mM imidazole concentration (Table 1). Sufficiently pure PepA was obtained, and the apparent size was 41 kDa on an SDS gel, matching well with the expected size of PepA with additional His-tag (Fig. 2. lane 3). The size of CY54 PepA is quite similar with those of other characterized LAB PepAs (41 kDa for *Lb. delbrueckii* subsp. *lactis* DSM 20072, 39.4 kDa for *L. lactis* subsp. *lactis* DSM20481, and 41 kDa for *L. lactis* subsp. *lactis* NCDO 712) [17, 24].

### Properties of Recombinant PepA

**Fig. 3:**
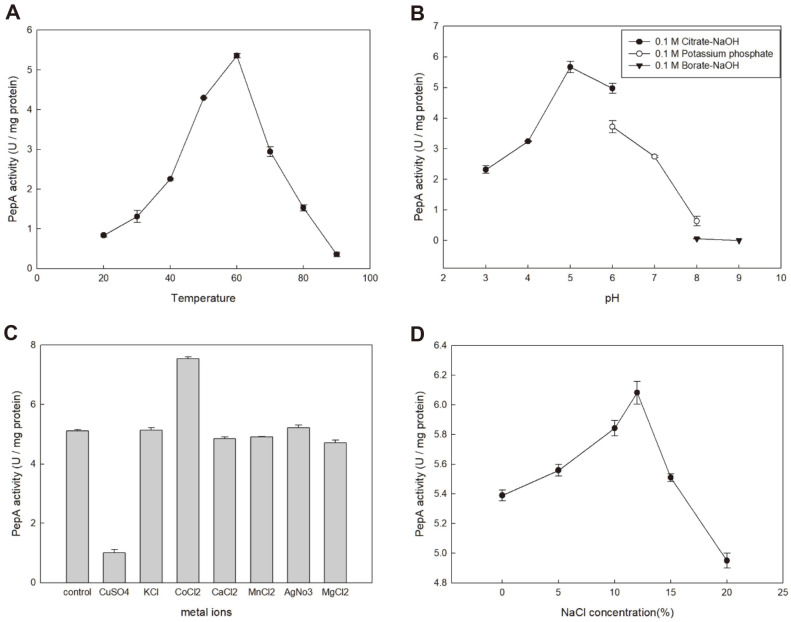
Properties of recombinant PepA under different conditions. Activities of recombinant PepA were measured at different conditions. (**A**) temperature, 20-90°C, (**B**) pH, 3-9, (**C**) metal ions, 2 mM each, and (**D**) NaCl concentration, 0-20%.

The optimal temperature of the recombinant PepA was 60°C. The activity sharply decreased at 70-80°C, and no activity was observed at 90°C (Fig. 3A). The optimum temperature of CY54 PepA was the same as that of *Lactobacillus delbrueckii* subsp. *lactis* DSM 20072 (60°C), lower than that of *Lactococcus lactis* subsp. *lactis* NCDO 712 (65°C), and higher than that of PepA from *Streptococcus cremoris* HP (50°C) (Table 2). The optimum pH for the recombinant PepA was 5.0 (Fig. 3B). Most PepAs are active at pH 6.0−8.0 as reported for those from *Lb. delbrueckii* subsp. *lactis* DSM 20072 (pH 6.0), *Lc. lactis* subsp. *lactis* DSM 20481 (pH 8.0) and *Lc. lactis* subsp. *lactis* NCDO 712 (pH 8.0) (Table 2). The effect of metal ions (2 mM) on the PepA activity was examined (Fig. 3C). The activity was increased by CoCl_2_ (152%), whereas the activity was strongly inhibited by CuSO_4_ (9%). Other metal ions did not significantly affect the PepA activity. The highest PepA activity was observed at 12% NaCl concentration, which was consistent with the optimum NaCl concentration for the growth of *T. halophilus* CY54 (Fig. 3D). The effects of various inhibitors and other reagents on the PepA activity were also investigated. SDS, the metallopeptidase inhibitor 1,10-phenanthroline and the metal chelator EDTA strongly inhibited PepA. There was little decrease in the activity by other reagents, indicating that PepA is a metallopeptidase (Table 3).

The substrate specificity of CY54 PepA was examined using 6 sets of *p*NA substrates. CY54 PepA strongly hydrolyzed Glu-*p*NA but did not hydrolyze Ala-*p*NA, Arg-*p*NA, Lys-*p*NA, Pro-*p*NA, and Met-*p*NA. The kinetic parameters of CY54 PepA were determined using Glu-*p*NA as the substrate. *K*m and *V*max were 0.63 ± 0.006 mM and 10.2 ± 0.02 U/mg protein, respectively, while Kcat was 0.0025 ± 0.000021/s, and *K*cat/*K*m was 3.97 ± 0.004/M/s (Table 2). The *K*m value of CY54 PepA was higher than those of PepAs from *Lc. lactis* subsp. *lactis* DSM 20481 [17] and *Lc. lactis* subsp. *lactis* NCDO 712 [24], but lower than that of PepA from *Lb. delbrueckii* subsp. *lactis* DSM 20072 [17].

### Heterologous Expression of *pepA* in *Weissella confusa*

Many *W. confusa* CB1 TFs were obtained by electroporation. The PepA activity of *W. confusa* CB1 harboring pHG240EpepA was measured (Table 4). *W. confusa* CB1 harboring intact pHG240E was used as a control. *E. coli* TF harboring pHG240EpepA showed 2.1 fold higher PepA activity than the control, DH5α cells harboring intact pHG240E. Likewise, *W. confusa* CB1 harboring pHG240EpepA showed 2.4 fold higher PepA activity than *W. confusa* CB1 harboring pHG240E. The results indicated that *pepA* from *T. halophilus* CY54 was successfully expressed in a heterologous host, and *W. confusa*, and *pepA* might be used to increase the proteolytic capacity of other LAB.

*pepA* from *T. halophilus* CY54 was cloned and overexpressed in *E. coli* BL21 (DE3). The properties of the recombinant PepA were examined. To date, while a few PepAs have been characterized and their properties reported, this is the first report on the characterization of PepA from the genus *Tetragenococcus*. CY54 PepA is not different significantly from other characterized LAB PepAs in terms of the size, optimum temperature, pH, and inhibition by inhibitors. However, a significant difference is the halophilic nature of CY54 PepA, as it shows the highest activity at 12% NaCl. No data are available for other PepAs. Considering its halophilic nature, CY54 PepA might play important roles in fermenting foods rich in proteins and with high salinities. CY54 PepA can probably be used for the production of flavoring hydrolysates from glutamyl/aspartyl-rich food proteins. Finally, while *T. halophilus* CY54 can be used as a starter for salted fish and fermented foods with high salt content, further research is needed to better understand the exact role and potential of CY54 PepA.

## Figures and Tables

**Table 1:** Purification of an aminopeptidase A from *T. halophilus* CY54.

Purification step	Total activity (U)	Total protein (mg)	Specific activity (U mg-1)	Purification fold	Yield (%)	Volume (ml)
Cell-free extract	68.3	122	0.56	1.0	100	10
His Trap FF Crude						
Elution 40	4.8	5.6	0.86	1.54	7.0	5
Elution 100	13.6	2.1	6.48	11.57	19.9	5

Elution 40 and 100: elution buffer containing 40 and 100 mM imidazole concentration, respectively.

**Table 2:** Characteristics of recombinant CY54 PepA and other PepAs.

Organism	Optimum Temperature (oC)	Optimum pH	Km (mM)	Inhibitors	Reference
*Tetragenococcus halophilus* CY54	60	5.0	0.63	1,10-phenantroline, EDTA	This study
*Streptococcus cremoris* HP	50-55	NR*	NR*	1,10-phenantroline, EDTA;DTT	[9]
*Lactobacillus delbrueckii* ssp. *lactis* DSM 20072	60	6	25.2	1,10-phenantroline, EDTA	[17]
*Lactococcus lactis* ssp. *lactis* DSM 20481	65	8	0.19	1,10-phenantroline, EDTA;DTT, β-mercaptoethanol	[17]
*Lactococcus lactis* ssp. *lactis* NCDO 712	65	8	0.28	EDTA	[24]

*not reported

**Table 3:** Effects of various inhibitors and reagents on the activity of CY54 PepA.

Reagents	Concentration (mM)	Relative activity (%)
Imidazol	40	81.8 ± 0.008
SDS	0.1	86.4 ± 0.001
	1.0	22.1 ± 0.002
	10	**0**
DTT	0.001	96.2 ± 0.005
	0.01	91.1 ± 0.006
	0.1	83.4 ± 0.001
EDTA	0.1	62.1 ± 0.001
	1.0	**0**
1,10-phenanthroline	0.1	91.2 ± 0.006
	1.0	79.7 ± 0.004
	10	**0**
PMSF	0.1	93.4 ± 0.006
	1.0	90.6 ± 0.006
	10	74.2 ± 0.002
Pepstatin A	0.001	97.5 ± 0.005
	0.01	96.8 ± 0.006
	0.1	91.9 ± 0.005
E64	0.001	92.7 ± 0.007
	0.01	84.6 ± 0.001
	0.1	80.2 ± 0.002

**Table 4:** Aminopeptidase A activities of *E. coli* and *W. confusa* transformed with pHG240EpepA.

Strains	Total activity (U/mg protein)
*E. coli* DH5α [pHG240E]	43.92 ± 0.32
*E. coli* DH5α [pHG240EpepA]	90.29 ± 0.14
*W. confusa* CB1 [pHG240E]	5.72 ± 0.03
*W. confusa* CB1 [pHG240EpepA]	13.63 ± 0.12
